# Genome-wide target profiling of *piggyBac *and *Tol2 *in HEK 293: pros and cons for gene discovery and gene therapy

**DOI:** 10.1186/1472-6750-11-28

**Published:** 2011-03-30

**Authors:** Yaa-Jyuhn J Meir, Matthew T Weirauch, Herng-Shing Yang, Pei-Cheng Chung, Robert K Yu, Sareina C-Y Wu

**Affiliations:** 1 Department of Biomedical Sciences, Chang Gung University, 259 Wen-Hwa 1st Road, Kwei-Shan, Tao-Yuan 333, Taiwan; 2 Banting and Best Department of Medical Research and Donnelly Centre for Cellular and Biomolecular Research University of Toronto, 112 College Street, Toronto, Ontario, M5 S 3E1, Canada; 3 YongKang Veterans Hospital, 427 Fuxing Road, Yongkang City, Tainan 71051, Taiwan; 4 Molecular Medicine Research Center, Chang Gung University, 259 Wen-Hwa 1st Road, Kwei-Shan, Tao-Yuan 333, Taiwan; 5 Institute of Molecular Medicine and Genetics and Institute of Neuroscience, Georgia Health Sciences University, 1120 15th Street, Augusta, Georgia 30912, USA

## Abstract

**Background:**

DNA transposons have emerged as indispensible tools for manipulating vertebrate genomes with applications ranging from insertional mutagenesis and transgenesis to gene therapy. To fully explore the potential of two highly active DNA transposons, *piggyBac *and *Tol2*, as mammalian genetic tools, we have conducted a side-by-side comparison of the two transposon systems in the same setting to evaluate their advantages and disadvantages for use in gene therapy and gene discovery.

**Results:**

We have observed that (1) the *Tol2 *transposase (but not *piggyBac*) is highly sensitive to molecular engineering; (2) the *piggyBac *donor with only the 40 bp 3'-and 67 bp 5'-terminal repeat domain is sufficient for effective transposition; and (3) a small amount of *piggyBac *transposases results in robust transposition suggesting the *piggyBac *transpospase is highly active. Performing genome-wide target profiling on data sets obtained by retrieving chromosomal targeting sequences from individual clones, we have identified several *piggyBac *and *Tol2 *hotspots and observed that (4) *piggyBac *and *Tol2 *display a clear difference in targeting preferences in the human genome. Finally, we have observed that (5) only sites with a particular sequence context can be targeted by either *piggyBac *or *Tol2*.

**Conclusions:**

The non-overlapping targeting preference of *piggyBac *and *Tol2 *makes them complementary research tools for manipulating mammalian genomes. *PiggyBac *is the most promising transposon-based vector system for achieving site-specific targeting of therapeutic genes due to the flexibility of its transposase for being molecularly engineered. Insights from this study will provide a basis for engineering *piggyBac *transposases to achieve site-specific therapeutic gene targeting.

## Background

DNA transposons are natural genetic elements residing in the genome as repetitive sequences. A simple transposon is organized by terminal repeat domains (TRDs) embracing a gene encoding a catalytic protein, transposase, required for its relocation in the genome through a "cut-and-paste" mechanism. Since the first discovery of DNA transposons in Maize by Barbara#McClintock in 1950 [1], transposons have been used extensively as genetic tools in invertebrates and in plants for transgenesis and insertional mutagenesis [2-7]. Such tools, however, have not been available for genome manipulations in vertebrates or mammals until the reactivation of a *Tc1/mariner*-like element, *Sleeping Beauty*, from fossils in the salmonid fish genome [8]. Since its awakening, *Sleeping Beauty *has been used as a tool for versatile genetic applications ranging from transgenesis to functional genomics and gene therapy in vertebrates including fish, frogs, mice, rats and humans [9]. Subsequently, naturally existing transposons, such as *Tol2 *and *piggyBac*, have also been shown to effectively transpose in vertebrates.

The Medaka fish (*Orizyas latipes*) *Tol2*, belonging to the *hAT *family of transposons, is the first known naturally occurring active DNA transposon discovered in vertebrate genomes [10]. *Tol2 *is a standard tool for manipulating zebrafish genomes and has been demonstrated to transpose effectively in frog, chicken, mouse and human cells as well [11]. Recent studies found that *Tol2 *is an effective tool both for transgenesis via pronuclear microinjection and germline insertional mutagenesis in mice [12]. Cabbage looper moth (*Trichoplusia ni*) *piggyBac *is the founder of the *piggyBac *superfamily and is widely used for mutagenesis and transgenesis in insects [13]. Recently, *piggyBac *was shown to be highly active in mouse and human cells and has emerged as a promising vector system for chromosomal integration, including insertional mutagenesis in mice and nuclear reprogramming of mouse fibroblasts to induced-pluripotent stem cells [14-19].

To date, most gene therapy trials have utilized viral vectors for permanent gene transfer due to their high transduction rate and their ability to integrate therapeutic genes into host genomes for stable expression. However, serious problems associated with most viral vectors, such as limited cargo capacity, host immune response, and oncogenic insertions (as evidenced by the retrovirus-based gene therapy) highlight an urgent need for developing effective non-viral therapeutic gene delivery systems [20,21]. Recently, *Sleeping Beauty*, *Tol2*, and *piggyBac *transposon-based vector systems have been explored for their potential use in gene therapy with proven successes [22-25]. However, for therapeutic purposes, a large cargo capacity is often required. The transposition efficiency of *Sleeping Beauty *is reduced in a size-dependent manner with 50% reduction in its activity when the size of the transposon reaches 6 kb [26]. *Tol2 *and *piggyBac*, however, are able to integrate up to 10 and 9.1 kb of foreign DNA into the host genome, respectively, without a significant reduction in their transposition activity [14,22]. Additionally, by a direct comparison, we have observed that *Tol2 *and *piggyBac *are highly active in all mammalian cell types tested, unlike *SB11 *(a hyperactive *Sleeping Beauty*), which exhibits a moderate and tissue-dependent activity [15]. Because of their high cargo capacity and high transposition activity in a broad range of vertebrate cell types, *piggyBac *and *Tol2 *are two promising tools for basic genetic studies and preclinical experimentation. Our goal here was to evaluate the pros and cons of *piggyBac *and *Tol2 *for the use in gene therapy and gene discovery by performing a side-by-side comparison of both transposon systems. In this study, we reported for the first time the identification of the shortest effective *piggyBac *TRDs as well as several *piggyBac *and *Tol2 *hotspots. We also observed that *piggyBac *and *Tol2 *display non-overlapping targeting preferences, which makes them complementary research tools for manipulating mammalian genomes. Furthermore, *piggyBac *appears to be the most promising vector system for achieving specific targeting of therapeutic genes due to a robust enzymatic activity of the *piggyBac *transposase and flexibility the transposase displays towards molecular engineering. Finally, results of our in-depth analyses of *piggyBac *target sequences highlight the need to first scrutinize the *piggyBac *favored target sites for the therapeutic cell type of interest before designing a customized DNA binding protein for fusing with the *piggyBac *transposase to achieve site-specific therapeutic gene targeting.

## Results

### Transposition activity of *piggyBac *and *Tol2 *in mammalian cells

With the ultimate goal of identifying and targeting safe sites in the genome at which to insert corrective genes, we previously explored three active mammalian transposases, *piggyBac*, *Tol2 *and *SB11 *(a hyperactive *Sleeping Beauty*) for their sensitivity to molecular modification [15]. After fusing the GAL4 DNA binding domain to the N-terminus of the three transposases, we only detected a slight change in the activity of the *piggyBac *transposase, whereas the same modification nearly abolished the activity of *Tol2 *and *SB11 *[15]. A recent genetic screen has yielded a novel hyperactive *Sleeping Beauty *transposase (designated as SB100X) that was shown to be more active than *piggyBac *under restrictive conditions that support their peak activity [28]. However, in this study we chose to focus on *piggyBac *and *Tol2 *but not *Sleeping Beauty *for the following reasons: (1) all of the reported attempts to modify the *SB11 *transposase either N- or C-terminally result in a complete elimination or a significant reduction in transposase activity; (2) *Sleeping Beauty *is more susceptible to over expression inhibition than *piggyBac *and *Tol2*; (3) the cargo capacity of *Sleeping Beauty *is limited; and (4) unlike *Tol2 *and *piggyBac *that are active in all mammalian cell types tested, *Sleeping Beauty *display cell-type dependent activity [15,27,34].

**Figure 1:**
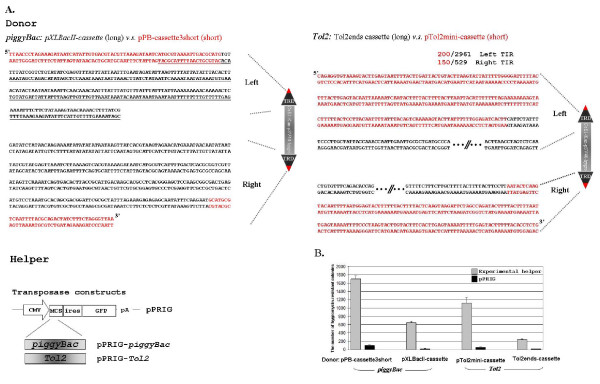
**Comparison of transposition activity between the long and short versions of *piggyBac *and *Tol2***. *A*. Donor and helper constructs of *piggyBac *and *Tol2 *used for the comparison. The activator sequence with enhancer activity in *D. melanogaster *is underlined. *B*. Transposition activity of the long vs. short version of *piggyBac *and *Tol2 *in HEK 293.

We have demonstrated that *piggyBac *and *Tol2 *display high transposition activity in several cell lines [15]. We now wish to explore the possibility of further enhancing their activity by trimming non-essential sequences from both transposons. Using a PCR-based strategy we generated pPB-cassette3short with the shortest TRDs reported replacing the long ones of the pXLBacII-cassette (Figure 1A) [29,30]. Similarly, based on the previous report, a new *Tol2 *donor, pTol2mini-cassette, with minimal terminal repeats [31] replacing the long ones of Tol2ends-cassette was also constructed (Figure 1A). The new helper plasmids of *piggyBac *(pPRIG-*piggyBac*) and *Tol2 *(pPRIG-*Tol2*) were also constructed by placing cDNA of *piggyBac *and *Tol2 *transposases, respectively, in the bi-cistronic transcriptional unit with GFP driven by the CMV promoter in the pPRIG vector (a gift kindly provided by Dr. Patrick Martin) (Figure 1A). To compare the transposition activity of the long versus short version of *piggyBac *and *Tol2*, the *piggyBac *or *Tol2 *donor with either long or short TRDs was co-transfected with its helper plasmid into HEK 293 cells. The transfected cells were subjected to a chromosomal transposition assay (as detailed in reference 32) to determine their transposition activity. Removing the majority of the terminal repeat sequences of *piggyBac *and *Tol2 *resulted in a 2.6- and 4.7-fold increase in transposition activity as compared to their wild-type counterparts (Figure 1B). Given that the sizes of the *piggyBac *and *Tol2 *donor plasmids are reduced by 1.75-and 1.4- fold, respectively, the observed increases in transposition activity for *piggyBac *and *Tol2 *are in effect 1.5- and 3.3-fold when normalized by the number of donor molecules transfected. True transpositions of pPB-cassette3short and pTol2mini-cassette in HEK 293 were further confirmed by retrieving chromosomal sequences flanking their target site (data not shown).

**Figure 2:**
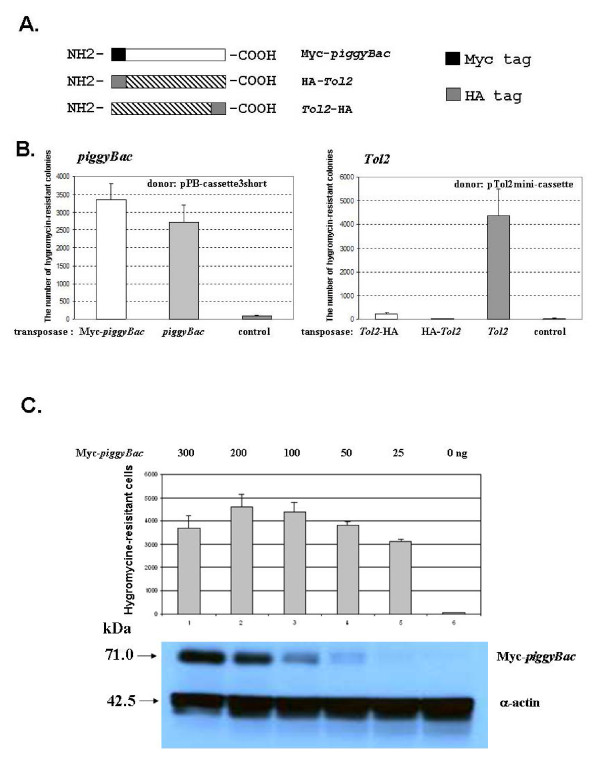
**Transposition activity of various engineered *Tol2 *and *piggyBac *transposases**. A. A schematic representation of tagged *piggyBac *and *Tol2 *transposases. B. The activity comparison between the wild-type and various epitope-tagged *Tol2 *and *piggyBac *transposases. C. The enzymatic activity of Myc-*piggyBac*. The enzymatic activity of Myc-*piggyBac *was measured under a fixed amount of pB-cassette3short (donor at 100 ng) co-transfected with increasing amounts of pCMV-Myc-*piggyBac *helpers (expressing the Myc-tagged *piggyBac *transposase) into HEK 293 cells. The lower panel depicts a Western blot indicating the expression level of the *piggyBac *transposase (detected by Myc antibody) and α-actin (detected by α-actin antibody) in the corresponding transfected cells. Western blotting was performed by isolating protein extracts from the remaining transfected cells of the same triplicate samples for the colony formation assay shown above.

In order to further explore their potential to be modified by molecular engineering, we Myc tagged the N-terminus of the *piggyBac *transposase (Myc-*piggyBac*) and HA tagged both the N- or C-terminus of the *Tol2 *transposase (HA-*Tol2 *and *Tol2*-HA) (Figure 2A). By co-transfecting pPB-cassette3short, and the helper plasmid expressing either wild-type or the chimeric *piggyBac *transposase into HEK 293 cells, we observed a slight increase in activity with the Myc-*piggyBac *as compared to its wild type counterpart (Figure 2B). An increase in activity after molecular modifications was also observed in several of our *piggyBac *chimeras including the GAL4-*piggyBac *which displayed a fluctuated activity that was sometimes higher than the wild-type *piggyBac *transposase (15 and Meir *et. al*., unpublished observations). Similar approaches, however, demonstrated that fusing the HA tag to either end of the *Tol2 *transposase almost completely eliminated its activity (Figure 2B).

To evaluate the activity of the *piggyBac *transposase, we then transfected a fixed amount of *piggyBac *donors (100 ng) with a various amount of helper plasmids bearing Myc-tagged *piggyBac *transposases (ranging from 50 to 300 ng) into HEK 293. *PiggyBac *transposition activity increases as the amount of *piggyBac *transposases increase until reaching its peak in cells transfected with 200 ng of helper plasmids (Figure 2C). As the amount of *piggyBac *transposases were reduced to the level barely detected by Western blotting, 68% of the transposition activity at its peak was still retained (compare lane 5 with lane 2 in Figure 2C), suggesting that *piggyBac *transposase is highly active.

### A global evaluation of *Tol2 *and *piggyBac *targeting preferences in the human genome

Genome-wide target profiling of *piggyBac *and *Tol2 *in the human genome has been reported recently [33-36]. However, all these studies were based on data sets obtained by retrieving chromosomal targeting sequences from a mixed population of transposon targeted cells or using a PCR-based strategy. To fully explore their potential as mammalian genome manipulation tools for gene therapy and gene discovery, reliable data sets of target sequence preferences based on targeting sequences retrieved form independent integrants are needed for genome-wide target profiling of *piggyBac *and *Tol2 *in the human genome. In this regard, as for piggyBac, we co-transfected pXLBacII-cassette and pPRIG-*piggyBac *into HEK 293 cells. Likewise, Tol2ends-cassette and pPRIG-*Tol2 *were co-transfected into HEK 293 for *Tol2*. The transfected cells were subjected to colony formation under hygromycin selection at a low density enabling for isolating individual colonies without cross-contamination (see Methods for details). Hygromycin-resistant colonies for *piggyBac *and *Tol2 *were individually cloned and further expanded. Genomic DNA isolated from individual clones was subjected to plasmid rescue for obtaining chromosomal DNA flanking the transposon insertion sites. We have isolated 164 and 114 individual colonies for *Tol2 *and *piggyBac*, respectively (Table 1). A total of 371 and 264 independent plasmids were respectively rescued from 142 *Tol2 *and 104 *piggyBac *colonies (Table 1) and subsequently sequenced. Only 149 and 315 of *piggyBac *and *Tol2 *targets resulted in a sequence of sufficient quality to execute a Blat search against the human genome database in the UCSC Genome Browser [37]. Among these, 107 *piggyBac *and 207 *Tol2 *targeting sequences had a strong match (over 95% sequence identity) to human genomic sequences (Table 1).

**Table 1:** The data sets of *piggyBac *and *Tol2 *genome-wide target profiling in HEK 293

Transposon	# of individual clones isolated	successful rate in plasmid rescue	# of individual plasmid rescued	# of targets with a quality sequence	# of targets mapped to the human genome*
***Tol2***	164	86% (142/164)	371	315	207
***piggyBac***	114	91% (104/114)	264	149	107

*: only the target sequences sharing ≥95% sequence identity with the corresponding human genome were selected

**Figure 3:**
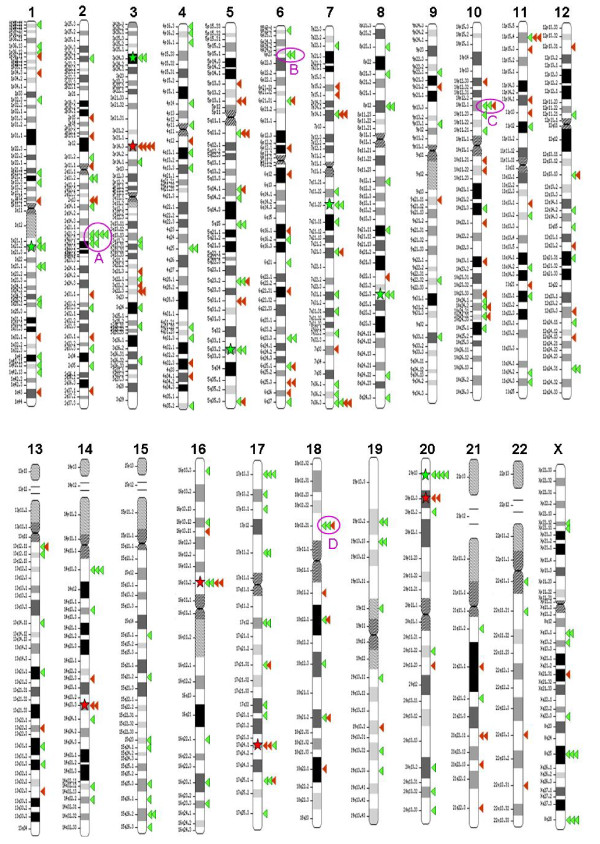
**Chromosome ideogram of target sites of *piggyBac *and *Tol2 *in HEK 293 The *piggyBac *and *Tol2 *targets are marked in red and green triangles, respectively**. The red and green stars label the site for *piggyBac *and *Tol2 *hotspots, respectively. Clusters (A-D) of *Tol2 *targets are circled in purple.

**Figure 4:**
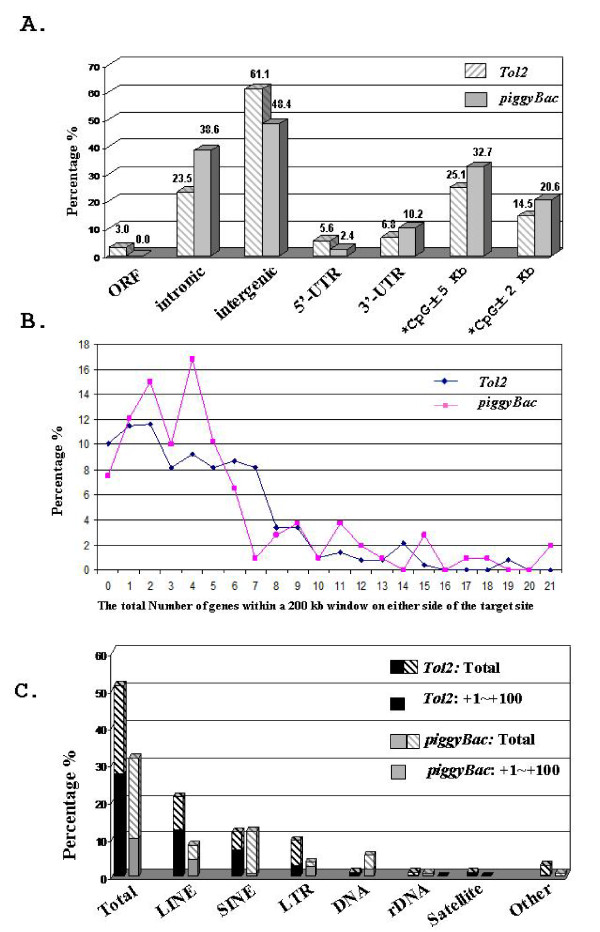
**Preferential target sites of *piggyBac *and *Tol2 *transposons in HEK 293**. A. The genome context of *Tol2 *and *piggyBac *target sites. *: both transposons tend to insert near CpG islands more often than expected by chance (Tol2, P < 10^-9^, piggyBac, P < 10^-11^; see Methods). B. The gene density around *Tol2 *and *piggyBac *target sites. C. Distributions of *piggyBac *and *Tol2 *target sequences in various types of repeats. Every bar represents the percentage of target sites found in the type of repeats indicated. The bottom part of each bar represents the percentage of targets located within at least 100 bp to the 3' end of the repeats targeted.

Based on the established data sets (Table 1), we performed target profiling of *piggyBac *and *Tol2 *in the HEK 293 genome. *Tol2 *and *piggyBac *display non-overlapping targeting profiles, with targets scattered over the entire genome (Figure 3). Although *Tol2 *targets were detected in all 23 human chromosomes (HEK 293 lacks the Y chromosome due to its female origin), no *piggyBac *targets were found in chromosome 15 (Figure 3). Interestingly, clusters of *Tol2 *targets within a 10 kb interval are often detected (four clusters as circled in purple in Figure 3 and supplementary 1), whereas no such clusters are apparent for *piggyBac. Tol2 *predominately targets intergenic regions (61.1%), whereas more than half of the *piggyBac *targets are located within known genes (51.6%) (Figure 4A). With respect to intragenic targeting preferences, both *piggyBac *and *Tol2 *favorably target the introns of known genes and no *piggyBac *target is found within the ORF of a gene. Regarding the target distribution in the UTR region, *piggyBac *displays a skew towards the 3' UTR, while no such bias can be seen in *Tol2 *(Figure 4A). Finally, consistent with previous reports [33-35], both *piggyBac *and *Tol2 *have a significant bias for integrating close to CpG islands, as compared to the computer simulated random integrations, with a higher bias detected in *piggyBac *than in *Tol2 *(Figure 4A).

To measure the distributions of *piggyBac *and *Tol2 *targets with regards to the gene density around the target sites, we counted the number of genes located within a 200 kb interval on either side of their target sites. By this analysis, *Tol2 *tends to target to regions with lower gene densities, particularly favoring regions with one to two genes located within a 200 kb window on either side of the insertion site (Figure 4B).

**Figure 5:**
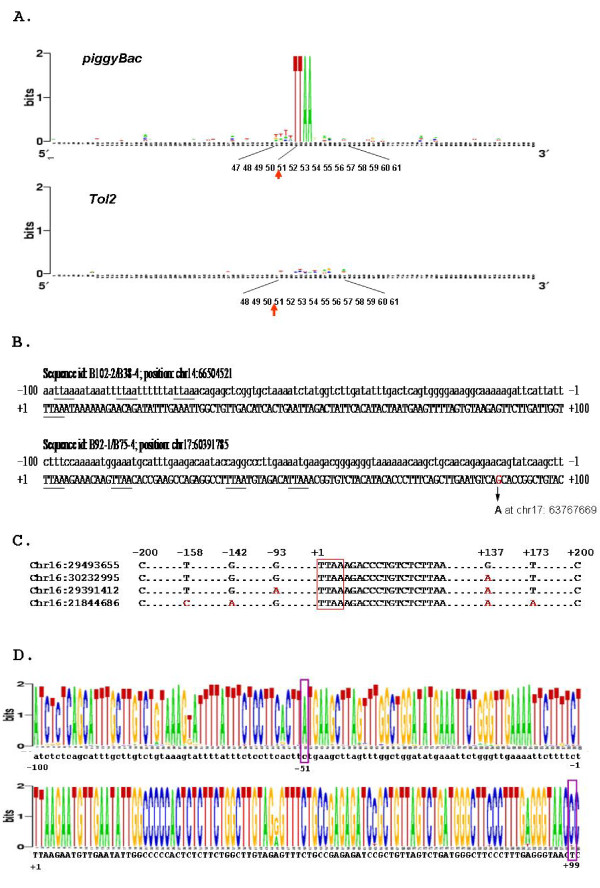
**In-depth analyses of *piggyBac *and *Tol2 *target sequence preferences in HEK 293**. A. The sequence logos of *piggyBa*c and *Tol2 *target sites. B. Two representative sequences flanking the sites repeatedly targeted by *piggyBac*. The TTAA tetranucleotide are underlined. C. A sequence alignment of four sequences on chromosome 16 that share 100% sequence identity with the first 100 bp of the *piggyBac *target B89-4. The residue that is different from the other three sequences at a given position is indicated in red. Dots represent all the primary sequences that are identical in all four sequences. The numbers on the top indicate the relative position of residues. D. The sequence logo of 184 sequences that share at less 97% sequence identity with the *piggyBac *target, B87-4. Note: The chromosomal sequence 5' and 3' to the target site are in lower cases and upper cases, respectively. *piggyBac *transposon is inserted at the position between -1 and +1.

We next determined the targeting preferences of *piggyBac *and *Tol2 *to different types of repeats in the human genome. Up to 51.2% of *Tol2 *targets were found within repeats, particularly LINEs (Figure 4C). The frequency of targeting to repeats by *piggyBac *was 31.8%, with a slight preference for SINEs. No *piggyBac *targets were detected in Satellite and rDNA. Repetitive sequences are stretches of DNA with similar sequences, and are found in numerous locations in the genome. It is possible that if one transposon displays a lower degree of sequence constraints for targeting than the other one, it may be able to target repeats more frequently than the other one. Based on this assumption and the fact that the sequences flanking the 3' end are significantly more important than that flanking the 5' end for both *piggyBac *and *Tol2 *target sites as determined by the sequence logo analysis detailed later (Figure 5A), we then applied sequence constraints to further address the targeting pattern of both transposons to different repeats. In this analysis, we only counted the inserts located at the site within and more than 100 bp upstream to the 3' end of targeted repeats (the lower part in each bar in Figure 4C). By applying this sequence constrain, the frequency of targeting repeats decrease much more dramatically in *piggyBac *than in *Tol2 *for the majority of repeat types (as compared the lower part of each bar to the whole bar in Figure 4C) suggesting that *piggyBac *may display a higher degree of sequence constrains than *Tol2 *in selecting their target sites.

### Sequence analyses of *Tol2 *and *piggyBac *target sites

To analyze the sequence preference for *piggyBac *and *Tol2 *targeting, we generated sequence logos for both transposon systems (Figure 5A). Consistent with previous reports, the characteristic TTAA tetranucleotide was exclusively found at the *piggyBac *target sites. Although no specific signature could be detected at *Tol2 *target sites, a weak but significant preference was observed in the first 10~11 bp 3' flanking the target site. Next, we searched for sites that are repeatedly targeted by either *piggyBac *or *Tol2*. Five and six sequences targeted repeatedly by *piggyBac *and *Tol2*, respectively, were identified (Table 2). And four out of 207 (1.9%) independent *Tol2 *targeting events occurred at the same position located within the intron of signal-regulatory protein delta (SIRPD).

**Table 2:** The *piggyBac *and *Tol2 *hotspots in the HEK 293 genome

Transposon	Target ID	Targeted sequence	Times	Position	Gene context	Targeted Gene	Near gene (distance bp)	Far gene (distance bp)
***piggyBac***	B87-5/B89-3	TTAAATAAAGATAATAATACTAACCATGGCA	2	3p14.3	INTRONIC	FLNB		
	B89-4/B77-4	TTAAAGACCCTGTCTCTTAAAAAAAAAAAAA	2	16p11.2	3'UTR	MLAS		
	B38-4/B102-2	TTAAATAAAAAAGAACAGATATTTGAAATTG	2	14q23.3	INTRONIC	GPHN		
	B71-1/B109-3	TTAAATTCCAGGTTTCTCAAAGAAAGCTTGT	2	20p12.3	INTERGENIC		BC043288(219296)	BMP2 (347908)
	B75-4/B92-1	TTAAAGAAACAAGTTAACACCGAAGCCAGAG	2	17q24.1	INTERGENIC		FLJ32065 (2686)	LRRC37A3 (101620)

								

***Tol2***	T47-2/T48-3	TGTTCCGCTCCTGGTGCGGGCCGAGACCCGG	2	1q21.2	INTRONIC	ZNF687		
	111-2/T119-1	TATGTGTAATAATGGAGGTATGTACAACAT	2	3p24.3	INTERGENIC		SGOL1 (385347)	HPX-42 (834010)
	T14-3/T17-1	AGAATAGGTATTTCTTTTTTTCTTCTTATC	2	5q33.2	INTERGENIC		C5orf3 (87465)	GRIA1 (92385)
	T2-1/T3-2	TCCACCACAGCATGAGTTAAACCAAAGTCT	2	7q11.23	INTERGENIC		POMZP3 (2680)	hPMSR6 (350693)
	T1-3/T4-1	CCTGCCCAGCTCGTAAAAGGATGCTCACCT	2	8q22.3	INTERGENIC		GRHL2 (694)	NACAP1 (122197)
	TB7-3/T113-1/T115-4/T120-2	AATTTATCCATTTCTTCTAGATTTTCTAGT	2	20p13	INTRONIC	SIRPD		

To further explore the nature of target site selection by *piggyBac *and *Tol2*, we performed a series of in-depth analyses on their target sequences. By conducting a Blat search against the UCSC genome browser database (assembly hg18) [37], we identified 16 *piggyBac *and 12 *Tol2 *targeting sequences which have at least the first 100 bp nucleotides 3' to the target site share more than 97% sequence identity with other sequences in the genome (Table 3). Surprisingly, 11 of the 12 *Tol2 *targets were located within repeats, but none of the 16 *piggyBac *targets was (Table 3). Again this observation may reflect a higher degree of sequence constrains in target site selection for *piggyBac *than for *Tol2*. Further analyses are required to reveal the nature of this discrepancy.

**Table 3:** The *piggyBac *and *Tol2 *targets located within the repetitive sequences of the HEK 293 genome

Transposon	Representative targets	Sequence (+1 ~ +30)	Position	Sequence identity			Types of repeats	Times targeted
						
				100%	99.9%~97.0%	Total		
***piggyBac***	B89-4	TTAAAGACCCTGTCTCTTAAAAAAAAAAAA	chr16(30140496)	4	0	4	NF	2
	B87-4	TTAAGAATGTTGAATATTGGCCCCCACTCT	chr3(55059477)	1	510	511	NF	1
	B75-4	TTAAAGAAACAAGTTAACACCGAAGCCAGA	chr17(60391785)	1	1	2	NF	2
	B85-4	TTAAAAAAGGCATTATTTTCGCAGCTATCT	chr6(125167100)	0	2	2	NF	1
	B42-3	TTAATTTACTTAAGATAATGGCCTCCACAC	chr22(15414966)	5	3	8	NF	1
	B100-1	TTAAGAAAGGAGTTGAATTAAGCTCAGGTT	chr1(120900969)	0	2	2	NF	1
	B90-1	TTAATATCCCACCTTTGCACAGTAGACAAT	chr3(137392502)	2	0	2	NF	1
	B92-2	TTAAACACACACTTAGAGGGAAATAATTCAT	chr18(11877795)	0	2	2	NF	1
	B82-3	TAAAGAATATAAGGCCAAGCACAGTGGCT	chr11(27402368)	1	1	2	NF	1
	B89-3	TTAAATAAAGATAATAATACTAACCATGGC	chr3(57972969)	1	1	2	NF	1
	B92-1	TTAAAGAAACAAGTTAACACCGAAGCCAGA	chr17(60391785)	2	2	4	NF	1
	B77-4	TTAAAGACCCTGTCTCTTAAAAAAAAAAAA	chr16(29401156)	0	4	4	NF	1
	B79-2	TTAAGGGGGGAAAACAGTTCAGGGCCAACA	chr14(55711916)	1	1	2	NF	1
	B84-1	TTAATGTTAAATTACAAACACTGTTTTATC	chr18(29434150)	0	2	2	NF	1
	B85-1	TTAAGCACAGTATCAGTGATAAAAATAGCT	chr1(201276407)	1	1	2	NF	1
	B82-1	TTAAGCTGAATCTGTTTTTCCCAGTGCCCC	chr2(231461932)	0	2	2	NF	1

								

***Tol2***	T111-3	TTTAAGAATGTTGAGTATTGGCCCCCACTC	chr8(9712455)	1	8	9	LINE	1
	T147-1	ATCCTGAGCAGCCGAATCTGCAATCATCTT	chr7(154665283)	1	1	2	LTR	1
	T3-2	TCCACCACAGCATGAGTTAAACCAAAGTCT	chr7(76097237)	2	0	2	LINE	1
	T88-3	GTCTGTACTGCTGCAAAGCTTCACAGACAG	chr10(98485313)	1	2	3	NF	1
	T115-4	AATTTATCCATTTCTTCTAGATTTTCTAGT	chr20(1472025)	0	3	3	LINE	4
	T103-2	GGCGCCCGCCACTACGCCTGGCTAATTTTT	chr21(13847935)	1	3	4	SINE	1
	T162-3	CCAGAGACCTTTGTTCACTTGTTTATCTGC	chr20(33206406)	202	ND	> 202	Low_complexity	1
	T157-1	CTCGTACGTAAGTTTTAGTGTGAACATATA	chr4(48956277)	4	3	7	LINE	1
	T157-2	GTTAACAGTGACCTATTTGGGAGAAGGGGA	chr7(66290199)	1	1	2	LINE	1
	T107-2	AATATATGAGTAGCTAAACAACTCTATAAG	chr2(97613844)	1	1	2	LINE	1
	T104-1	GAACACATGGACACAGGAAGGGGAACATCA	chr3(134171282)	1	52	53	LINE	1
	T25-3	ACCCCATCTCTACTAAAAATACAAAAAATT	chr6(166733349)	1	1	2	SINE	1

To study the nature of *piggyBac *target specificity, we next examined the neighboring sequences around five *piggyBac *hotspots. We observed that several TTAA tetranucleotides are located within a 100 bp interval of two *piggyBac *hotspots. The target sequences in B102-2 and B38-4 are identical and contain three TTAA tetranucleotides within a 100 bp interval upstream of the actual *piggyBac *TTAA target (Figure 5B). Similarly, the sequence of another *piggyBac *hotspot (as in B92-1 and B75-4), contains three TTAA tetranucleotides within the 100 bp interval downstream of the genuine TTAA *piggyBac *target site. A Blat search has identified another sequence which is located 3.3 Mb away and shares 99.5% sequence identity with the target site of B92-1 and B75-4. As detailed in the lower sequence of Figure 5B, a G (in red) to A substitution is identified at +88 on the other sequence where the *piggyBac *target site is designated as 0.

The fact that *piggyBac *targeted repeatedly to the same TTAA but not the adjacent TTAA tetranucleotides or to the TTAA site on another highly identical sequence nearby raise the possibility that the genuine TTAA *piggyBac *targets may be determined by some intrinsic sequence constraints flanking the target site. To further address this possibility, we focused on two other *piggyBac *target sequences, the B89-4 and B87-4 (Table 3). By a Blat search, we identified four sequences on chromosome 16 that share 100% sequence identity with one of the *piggyBac *hotspot as in B89-4 and B77-4 (Table 3). We then performed a multiple sequence alignment on these four sequences. Although the primary sequence of these four sequences with a 200-bp interval on either side of the TTAA target site is almost identical, both B89-4 and B77-4 target to the same TTAA tetranucleotide on the top but not the other three similar sequences in Figure 5C. Another example, B87-4, was found to share at least 97% sequence identity with 510 sequences elsewhere in the human genome, yet none of these highly similar sequences were targeted by *piggyBac *(Table 3). To gain further insight into the nature of *piggyBac *target selection, we retrieved the top 184 sequences that share 99% sequence identity with the first 100 bp of the B87-4 target. As revealed by the sequence logo analysis, the primary sequence of these 184 sequences is highly conserved (Figure 5D). By designating the first T of TTAA as +1, the conserved A at -51 and C at +99 are changed to C and T, respectively, in the B87-4 target. Collectively, these observations strongly suggest that *piggyBac *does not target arbitrarily to any TTAA tetranucleotide in the human genome but rather to the TTAA sites in a specific sequence context.

### The activity of genes nearby the *piggyBac *and *Tol2 *hotspots

**Figure 6:**
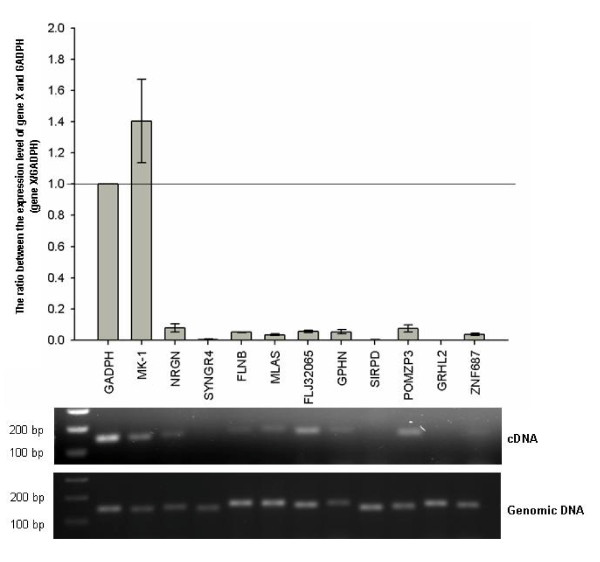
**The activity of genes which are close to or located at the site repeatedly targeted by *Tol2 *or *piggyBac***. The upper panel is a histogram showing the ratio of gene expression level between the housekeeping gene, GAPDH and the gene of interest that is either repeatedly targeted by *piggyBac *or *Tol2*, or is located within a 10-kb interval of *piggyBac *or *Tol2 *hotspots as measured by Q-RT-PCR. A set of neural genes (MK-1, NRGN, and SYGR4) with a high level to no expression in HEK 293 cells is also served as references. The lower panel is a DNA-agarose gel image of a representative Q-RT-PCR reaction showing the PCR products at the end of the 30^th ^cycle. Genes targeted repeatedly by *piggyBac*: FLNB; GPHN; and MLAS. Genes near the *piggyBac *hotspot: FLJ32065. Genes targeted repeatedly by *Tol2*: SIRPD and ZNF687. Genes near the *Tol2 *hotspot: POMZP and GRHL2.

Genome-wide targeting analyses of retroviruses have revealed their biased nature in preferentially targeting to active regions of the host chromatin. To address whether gene activity had an influence on target preferences of *piggyBac *and *Tol2*, we performed quantitative RT-PCR (Q-RT-PCR) analyses, focusing mainly on genes located within or within a 10 kb interval from either *Tol2 *or *piggyBac *hotspots. The house keeping gene GAPDH and three neural genes with a broad range of expression levels in HEK 293 [38] were selected to serve as references for Q-RT-PCR analyses. It is impossible to assess the relative abundance of difference genes by directly comparing the Q-RT-PCR signal between various primer pairs. Hence, we designed the primer pair within the same exon for each gene. The expression level for each gene was then evaluated by the ratio of the relative copy number derived from Q-RT-PCR and that derived from quantitative PCR (Q-PCR) by using the same primer pair on mRNA and the genomic DNA of HEK 293, respectively. Most of the genes tested were either not expressed or expressed at a much lower level as compared to GADPH (Figure 6). Notably, SIRPD, the gene containing the most frequently targeted *Tol2 *hotspots was barely expressed in HEK 293 (Figure 6). Hence, it is highly likely that gene activity has no influence on the hotspot selection of *piggyBac *and *Tol2*. Indeed we have recently identified a *piggyBac *hotspot located at a gene that is silenced in HEK 293 (Meir et. al., unpublished observation).

### Risk assessment of targeting within or near cancer-related genes by *piggyBac *and *Tol2*

**Figure 7:**
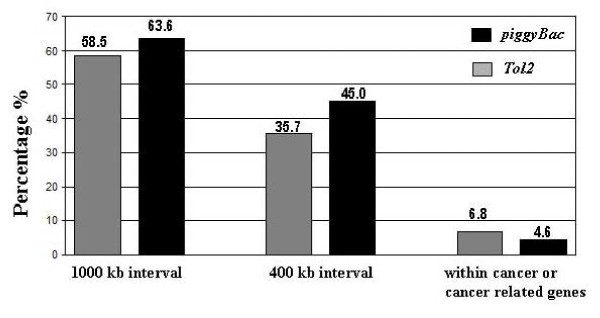
**A risk evaluation of *piggyBac *and *Tol2 *for targeting to the region located within or near cancer related genes in a genome-wide scale**. The histogram shows the percentage of *piggyBac *or *Tol2 *targets located within or within a defined distance away from cancer-related genes.

Random insertion mutagenesis is a real threat to gene therapy [39]. The mutagenic potential caused by random insertions of any transposon remains the greatest concern for their advancement to clinical applications. In this regard, we assessed the risk of *Tol2 *and *piggyBac *for their potential of inducing oncogenesis by counting the number of *piggyBac *or *Tol2 *targets located either directly within or within a defined distance of a cancer-related gene. The frequency of targeting to sites within either a 400-kb or 1000-kb distance from cancer-related genes was significantly higher in *piggyBac *than in *Tol2 *(Figure 7). However, the frequency of targeting within a cancer-related gene was higher in *Tol2 *than in *piggyBac *(Figure 7). Cancer related genes targeted by *Tol2 *or *piggyBac *are listed in Table 4. Notably, *piggyBac *targeted twice to the same site within one particular cancer-related gene, gephyrin (GPHN), raising a great concern for its safe use in gene therapy [40-42].

**Table 4:** A list of cancer-related genes targeted by *iggyback *or *Tol2*

Transposon	Target ID	Sequence	Targeted Cancer Gene	Annotation
***piggyBac***	B102-2	TTAAATAAAAAAGAACAGATATTTGAAATTGGCTGTTG	GPHN	gephyrin
	B107-3	TTAATGATTCTTTCCATTTCTTTTATTCTTTTCCTAGC	HHEX	hematopoietically expressed homeobox
	B27-3	TTAATAGAAAGGAAGGGACCATGTTTAACATAAATGCT	POU6F2	POU class 6 homeobox 2
	B38-4	TTAAATAAAAAAGAACAGATATTTGAAATTGGCTGTTG	GPHN	gephyrin
	B63-1	TTAAGTTTTCAGTGGCTGAAAGTTGGCAGTCTGAAAAA	ARF4	ADP-ribosylation factor 4
	B81-1	TTAAGTGCTTTTGGCTGTTTTCCCAAACATCCAGACAT	SMAD5	SMAD family member 5

				

***Tol2***	T12-2	GGTAGGAGTTATCTGAGTCAGGCCTGCCCTTGGCTTGG	SPECC1	cytospin B
	T121-1	CTCCTGGGTGACCCTCGCCTGAGCCTCCTGGCCCTTCC	RAB40B	RAB40B, member RAS oncogene family
	T102-4	GACACAAACACACACATGCTATACCTTTGTATTACACT	TCF4	transcription factor 4
	T124-4	GCGGCTGTCCTCCAGCAACAGGTGCACATTCCCGGGCT	TNK1	tyrosine kinase, non-receptor
	T130-2	CAAATAAATGAATGTTATGAATTTTTGAGGGTAGGAAA	SEMA3C	sema domain, immunoglobulin domain (Ig), (semaphorin) 3C precursor
	T137-3	AAAGAGAGGCCCAATCCTGTGGAGTGAGTCACTGGGGG	ALPL	alkaline phosphatase, liver/bone/kidney
	T137-4	ATTTTCTGTCTGCTCTTTGGTCACTTCCCATTCTTTTT	PARD3	par-3 partitioning defective 3 homolog (C. elegans)
	T157-4	TTTATTTGTCCTGCACTTATGAAGCATAGTTTGGCAGG	PGR	progesterone receptor
	T165-1	GAAACCGGCGAAAAGGTTAGCTGTCGCTGGCTAGTATT	RASSF3	Ras association (RalGDS/AF-6) domain family member 3
	T22-1	TTCCTAAGCTACAATAAACCACATATGAAAAACTAAAG	HD	huntingtin
	T26-1	AGCCTGAGTAAAATAGTGAGACTCTGTTTCTGCAAAAC	LRP1B	low density lipoprotein-related protein 1B
	T43-3	TCTGGAAGGTGAGGCAGACGTGCCCACCGCCTCCATGC	HLXB9	motor neuron and pancreas homeobox 1
	T91-4	TTCAGGGGGTGTGTTGGAGGGGAATCGCCGGCCTGCCT	IHH	Indian hedgehog homolog (Drosophila)
	TB7-5	AATTTATCCATTTCTTCTAGATTTTCTAGTTTATTCGC	FKBP1A	FK506 binding protein 1A, 12kDa
	TB70-1	GTGCACACACTCACTCTCTCTTTCTCCTTCAGATAATA	FOXP1	forkhead box P1
	TB77-2	CCCCTCACCCTCGGACCCTTCACCGCGACCCCCGCGCC	RANBP9	RAN binding protein 9
	TB81-1	TGCAGTACAGTGCGGGGGGAAAAAAACAACAGCAAAAG	EGF	epidermal growth factor (beta-urogastrone)

## Discussion

The longer the foreign sequences introduced into the host genome, the greater the probability of evoking adverse consequences, such as transgene silencing and dysregulation of the endogenous genes nearby. Hence, for both basic research and clinical applications, a transposon system with smallest terminal repeats for genetic manipulations is desired. By removing most of the nonfunctional sequences of *piggyBac *and *Tol2 *TRDs, we observed a 1.5- and 3.3-fold increase in transposition activity for *piggyBac *and *Tol2*, respectively. The increase in transposition activity for both *piggyBac *and *Tol2 *is unlikely to be due to their reduction in size, since the *piggyBac *element in the pXLBacII-cassette and the *Tol2 *element in the Tol2ends-cassette are both within their maximal cargo capacity of 9.1 Kb and 10 Kb, respectively [14,22]. In general, the transposition activity of a transposon negatively correlates with the fitness of the host. Although in most cases the activity of transposons in the host is abolished due to mutations and deletions, some transposons are intact but are completely silenced epigenetically by host defense mechanisms [43]. For example, RNAi is the mechanism for silencing the *Tc1 *DNA transposon in the germ line of *Caenorhabditis elegans *[44]. Unlike pXL-BacII-cassette only consisting of 245 bp-left and 313 bp-right TRD, the *Tol2*end-cassette preserves most of the non-coding *cis *sequences of the wild-type *Tol2 *transposon. These "non-essential sequences" may be susceptible to epigenetic silencing and in turn attenuate their transposition activity. This possibility may explain why extra *cis *sequences in Tol2ends-cassette has a greater impact in deregulating transposition activity than that of pXLBacII-cassette. This observation further implicates the possible interaction between epigenetic silencing factors and the *cis *sequence of wild-type transposons, and for *Tol2 *in particular. Studies are now underway to address this possibility.

Unlike our findings that pPB-cassette3short with short TRDs at the ends results in a higher activity than its long counterpart in HEK 293, attempts to transform *D. melanogaster *with p(PZ)-Bac-EYFP consisting of 35-bp 3'TRD and 63-bp 5'TRD yielded transformation frequencies far less than full-length *piggyBac *constructs (reduced from 15% to 0.6%) [29]. This discrepancy may simply reflect the differences in the components and/or the mechanism involved in transposition between mammalian and insect cells. It is also possible that the extra 5 and 4 nucleotides included in our 3'- and 5'-TRD, respectively, are crucial for an effective transposition. Another important feature of our functional *piggyBac *terminal sequences (referred as micro-PB hereafter) is that most of the activator sequences (as underlined in Figure 1A) identified previously in *D. melanogaster *[45] are excluded. In this respect, the micro-PB may potentially be a safer cis-*piggyBac *element as a mammalian genetic tool as compared to the minimal *piggyBac *cis-sequence identified previously. Studies are now underway to address whether micro-PB exhibits any enhancer or silencer activity.

Genome-wide targeting profiles of *piggyBac *and *Tol2 *in the human genome have been previously reported [31-34]. All of these analyses utilized chromosomal target sequences that were retrieved either by plasmid rescue from a heterogenous population of targeted cells or by PCR-based strategies using a limited amount of genomic DNA isolated from individual targeted clones grown on 96-well plates. Several factors may introduce strong biases into the data sets obtained in these studies including (1) differences in proliferation rates of the individual targeted cells, (2) intrinsic difficulties in retrieving certain targeting sequences, and (3) biases in obtaining PCR products from certain templates but not from the others. Hence, to fully evaluate the pros and cons of *piggyBac *and *Tol2 *for gene discovery and gene therapy, a direct comparison of their genome-wide targeting profile based on reliable data sets obtained within the same experimental setting was needed. To achieve this goal, we utilized a labor intensive strategy involving isolating, expending, and performing plasmid rescue to retrieve chromosomal targeting sequences for each individual HEK 293 clone targeted. Based on the following observations, we believe the data sets established in this study provides reliable insights into the targeting profiles of *piggyBa*c and *Tol2*. First, we successfully rescued plasmids from 87% and 91% of *piggyBac *and *Tol2 *targeted clones, and the majority of clones that were not rescued were due to a lack of sufficient genome DNA for performing plasmid rescue. Second, several copies of an identical plasmid were often obtained in the same targeted clones, suggesting that most, if not all, inserts in the same clones were successfully recovered. Third, for each individual clone targeted, we normally obtained 1-4 different inserts, consistent with a recent report that the copy number of *Tol2 *and *piggyBac *in HeLa cells ranges between 1-3 and 1-4, respectively [34]. Identifying targeted sites in individual clones has led to the identification of *piggyBac *and *Tol2 *hotspots and allowed us to perform a detailed and unbiased analysis on target site preferences for both transposon systems.

All *piggyBac *and *Tol2 *hotspots identified in this study are likely to be *bona fide *given the following reasons. First, the protocol (as detailed in the methodology section) used to isolate individual targeted clones is intentionally designed to avoid cross-contamination between individual drug-resistant colonies. Second, all of the target sequences in this study were retrieved using plasmid rescue rather than a PCR-based strategy. A small amount of contaminating genomic DNA, if any, is not sufficient for a successful plasmid rescue. Third, the four *Tol2 *targets mapped to the hotspot located in the SIRPD locus were derived from two separate experiments suggesting the occurrence of independent targeting events at this particular site in the HEK 293 genome. Finally, all of the *piggyBac *and *Tol2 *clones with a hotspot targeted contain additional integrations mapped to distinct chromosomal locations (data not shown), indicating all of these targeted clones were indeed independent. Our analyses of *Tol2 *have revealed a distinct global targeting distribution among 23 human chromosomes in HEK 293, which stands in sharp contrast to the reported *Tol2 *distribution in HeLa cells (compare Figure 4 with the Figure 6A in reference 34). Distinct *Tol2 *genome-wide targeting profiles in HEK 293 and HeLa cells seem to reflect their difference in frequency of targeting to different genomic contexts. For instance, our analyses revealed 23.5% and 15.4% of *Tol2 *intronic and exonic targeting frequency in HEK 293, respectively (Figure 5A), while the reported intronic and exonic targeting rate of *Tol2 *in HeLa cells are 45.1% and 3.5%, respectively (Table 2 in reference 34). Discrepancies in the frequency of *Tol2 *targeting to various repeat types between our study and others were also detected. Two factors may account for the observed discrepancies: namely (1) differences in strategies, and (2) differences in *Tol2 *targeting preferences in HEK 293 and HeLa cells. The former factor should not substantially contribute to the great difference in targeting preferences seen in the two separate studies, since even if one approach is less biased than the other, a certain degree of overlapping in *Tol2 *target distributions should still be detected in both human cell types. However, this is not the case. Hence, the non-overlapping *Tol2 *target profiles are likely due to differences in cell types. As for *piggyBac*, although its intragenic target rate in this study (51.6% in HEK 293) and in other studies (51.9% in primary T cells) is similar, we observed a much higher frequency of *piggyBac *targeting to untranslated regions in HEK 293 (15.8% total) than what was observed in primary T cells (1.7% total) (compare Figure 5A with data reported in reference 35). Additionally, we fail to detect any *piggyBac *targets that are found both in HEK293 (this study) and in human T cells [35]. Unlike the data set established in this study, the genome-wide *piggyBac *targets in primary T-cells were obtained from a heterogenous population of *piggyBac *targeted clones [35]. Consequently, the data set obtained from primary T-cells is inevitably biased to the target sites that are easily retrieved by plasmid rescue, a factor that may contribute significantly to the sharp contrast in the targeting profiles of *piggyBac *observed in the two different cell types. However, our data set revealed five *piggyBac *hotspots in HEK 293 and yet no target in our data set is found in that of primary T cells, suggesting cell type differences may still be the major contributing factors when explaining these observed differences. Furthermore, these differences were likely to be amplified by the fact that unlike T-primary cells which contain normal 46 chromosomes, HEK 293 is a transformed cell line with an aberrant karyotype of 64 chromosomes as characterized originally. Collectively, comparisons of our data with that of others highlights the necessity for (1) obtaining a reliable data set for genome-wide target analyses (preferably by retrieving all target sequences for each individual targeted clone) and (2) re-evaluating the genome-wide target profile of transposons (at least *piggyBac *and *Tol2*) in the specific stem cell type of therapeutic interest before advancing them to clinical uses.

The reliable data sets obtained in this study allow us to perform in-depth sequence analyses of their targets without ambiguity. The sequence logo of *Tol2 *detected subtle but significant information present within the first 11 base pairs on the 3' end of *Tol2 *target sites. Furthermore, as indicated in Table 3 despite the fact that the target sequence of the most frequently targeted *Tol2 *hotspot (4 out of 207) is actually located within LINEs and shares more than 97% sequence identity with two other sequences in the genome, *Tol2 *only targeted to this particular site but not to other similar sequences. Collectively, these observations strongly suggest even though no distinct features of *Tol2 *target sequences can be readily identified, *Tol2*, like *piggyBac*, also targets in a selective manner in the host genome. The in-depth sequence analyses also revealed the following important features of *piggyBac *targeting preference: (1) TTAA sites in a particular sequence context are targeted by *piggyBac*, as opposed to arbitrary TTAA sites, (2) there is no direct correlation between *piggyBac *hotspots (and *Tol2 *hotspots as well) and the activity of genes either contained within or near the hotspots, and (3) at least the first 100 nucleotides on either side of *piggyBac *target site seem to be important for *piggyBac *target selection, and a subtle change in the primary sequence within this 200 bp interval may result in losing its potential for *piggyBac *targeting. These insights will provide a solid knowledge basis for engineering *piggyBac *transposase to achieve site-specific therapeutic gene targeting.

Powerful genetic tools enabling the probing of functions of both coding and non-coding genome sequences are urgently needed to facilitate the progress in determining the genetic factors that contribute to our uniqueness as human beings in a post-genomic era. The fact that *piggyBac *favorably targets intragenic chromosomal regions makes it a great tool for uncovering the functions of protein coding genes. Transposable elements are often considered "junk" DNA in the human genome. An increasing body of evidence, however, suggests that a fraction of these repetitive sequences are active and play import roles in epigenetic gene regulation [43,44,46,47]. The preference of *Tol2 *to target genomic repeats makes it an ideal tool for revealing new functions of transposable elements residing in our genome. Collectively, the non-overlapping genome-wide target profiles of *piggyBac *and *Tol2 *potentially makes them complementary research tools for studying the human genome.

Genotoxicity caused by a single integration event mediated by the retrovirus-based vector has resulted in the development of T-cell leukemia in 5 of 20 patients treated for SCID with one death reported [39]. Hence, no wild type DNA transposon is considered safe for gene therapy since they all introduce transgenes into a host genome in a random fashion. Indeed, our genome-wide target profiling of *piggyBac *in HEK 293 revealed a *piggyBac *hotspot located within the coding region of gephyrin, a scaffold protein implicated in colon cancer and adult T-cell leukemia [40-42]. Most active mammalian genome manipulating enzymes, including viral integrases and DNA transposase, must therefore be molecularly modified to achieve the ultimate goal in gene therapy: targeting the therapeutic gene into a predetermined genomic site where the therapeutic gene can be stably and faithfully expressed without disturbing the global gene expression profile. Put into perspective, *piggyBac *is by far the most promising vector system for gene therapy, as *piggyBac *transposase is the only one capable of being molecularly modified without substantially losing activity (reference 15 and this study).

## Conclusions

The transposon-based tool box for mammalian genomic manipulations is expanding. Here, we engaged in a side-by-side comparison of two highly effective mammalian active transposons, *piggyBac *and *Tol2*, to evaluate their pros and cons for gene discovery and gene therapy. We report the identification of the shortest *piggyBac *TRDs, micro-PB, which have a higher transposition efficiency in HEK 293 than that of the previously reported *piggyBac *minimal terminal repeat domains, mini-*piggyBac*. Our genome-wide target profiling reveals that *piggyBac *and *Tol2 *display complementary targeting preferences, making them suitable tools for uncovering the functions of protein-coding genes and transposable elements, respectively, in the human genome. Our results suggest that *piggyBac *is the most promising DNA transposon for gene therapy because its transposase is likely the most amenable mammalian genetic modifier for being molecularly engineered to achieve site-specific therapeutic gene targeting. Our in-depth sequence analyses of *piggyBac *targets revealed that the sequence context near and within a considerable distance from the TTAA *piggyBac *target site is highly important in site selection. Based on this observation, it is clear that in order to advance *piggyBac *for a clinical use in gene therapy, a safe and favorable site for *piggyBac *targeting in the genome of the appropriate therapeutic stem cell should first be identified, followed by the engineering of *piggyBac *transposase to achieve site-specific gene targeting.

## Methods

### Transposon constructs

The plasmid construction described in this study followed the protocol of Molecular Cloning, 3^rd ^edition, CSHL [48]. The sequences of all constructs involving PCR-based cloning were confirmed by DNA sequencing. The process of each construction is described briefly as follows:

#### (1) pPB-cassette3short

The short *piggyBac *TRDs (i.e. 746~808 3' LTR and 1426~1460 5' LTR as in pXL-BacII [29,30]) were obtained from the PCR mixture consisting of the following four pairs of primers; pB-11-KpnI (atcgggtaccttaaccctagaaagataatcatattg), pB-5-forward (ggtaccCCCTAGAAAGATAATCATATTGTGACGTACGTTAA AGATAATCATGCGTAAAATTGACGCATGctcgag), pB-6-reverse (gagctcCCCTAGAAAGATAGTCTGCGTAAAATTGACGCATGccaccgcggtggatttaa atctcgagcatgcgtca), and pB-12-SacI (cgatgagctcttaaccctagaaagatagtctgcg). The resulted amplicon containing both 67 bp 5' and 40 bp 3' TRD with SwaI and Xho I restriction sites in between was cloned into pBS-SKII through Kpn I and Sac I restriction sites to obtain the pPBendAATT. The same cassette (containing the hygromycin resistant gene driven by SV40, the replication origin, ColE1, and the kanamycin resistant gene) as in pXLBacII-cassette [15] was inserted between short *piggyBac *TRDs in pPBendAATT through the blunt-ended Xho I site to make the intermediate construct, pPBcassette3. To generate the pPB-cassette3short, pPBcassette3 was digested with Acc65 I and Afl III to remove the ampicillin resistant gene and the f1 replication origin. The remaining DNA fragment was blunt-ended followed by self-ligation to generate the final construct, pPB-cassette3short.

#### (2) pTol2mini-cassette

To construct the Tol2 donor with short TRDs, two separated PCR products were generated by two sets of primers, Tolshort-1 (atcgggtaccatttaaatCAGAGGTGTAAAGTACTTG)/Tolshort-2 (tatcaagcttagatctagAAGTGATCTCCAAAAAATAAG) and Tolshort-3 (ctaagcttgatatcaacggatccAATACTCAAGTACAATTTTAATGG)/Tolshort-4 (cgatgagctcatttaaatCAGAGGTGTAAAAAGTACTC), respectively using the Tol2end-cassette [15] as a template. Next, these two PCR products were served as templates to produce the third PCR product using the Tolshort-1 and Tolshort-4. The third PCR product was cloned into the Kpn I and Sac I site of pBS-SK II vector to generate the miniTol2-end. The same cassette as described in section (1) above was then inserted into the EcoR V site of miniTol2end to generate pTol2mini-cassette.

#### (3) pPRIG-*piggyBac*

To generate pPRIG-*piggyBac*, the coding sequence of the *piggyBac *transposase was PCR amplified from pcDNA3.1Δneo-*piggyBac *[15] using primer piggyBac-10 (ATCGGAATTCACCATGGGTAGTTCTTTAGACG) and primer piggyBac-11 (AAGGCACAGTCGAGGCTG). The PCR product was cloned into the EcoR I and Not I site of the pPRIG vector.

#### (4) pPRIG-*Tol2*

The coding sequence of the *Tol2 *transposase was obtained from the Xba I/BamHI restriction fragment of pcDNA3.1Δneo-Tol2 [15] and then inserted into the Stu I and BamHI sites of pPRIG vector.

#### (5) pCMV-Myc-*piggyBac*

The same fragment containing the ORF of *piggyBac *transposase as described in section (3) above was cloned into the pCMV-myc vector (Clontech, Inc) to generate pCMV-Myc-*piggyBac*.

#### (6) pPRIG-HA-*Tol2*

A pair of complementary oligos containing the sequence of the HA tag was synthesized, annealed and inserted into the BamHI site of pPRIG-*Tol2 *vector to generate pPRIG-HA-*Tol2 *which expresses a N-terminal HA tagged *Tol2 *transposase. The clones with a correct orientation were obtained and verified by DNA sequencing.

#### (7) pPRIG-*Tol2*-HA

pPRIG-*Tol2*-HA expressing the C-terminal HA tagged *Tol2 *transposase was constructed by swapping the restriction fragment of XcmI and SphI of pCR4-TOPO-Tol2HAc (the detailed procedure regarding the construction of this plasmid is upon requested) with those in pPRIG-*Tol2*.

### Cell culture and transposition assay

HEK 293 cells were maintained in MEMα medium (HyClone) supplemented with 10% FBS (HyClone), 100 units/ml penicillin, and 100 μg/mL streptomycin. The details for the transposition assays were described previously [32].

### Activity assay of the *piggyBac *transposase

A similar procedure as detailed previously [32] was used to co-transfect 100 ng of *piggyBac *donor, with various amount of the *piggyBac *helper, pCMV-Myc-*piggyBac*, ranging from 0 - 300 ng into 1.2 × 10^5 ^of HEK 293 cells. pcNDA3.1ΔNEO, an empty vector used in our previous study (Wu et.al 2006), was used to top the total amount of DNA transfected to 400 ng. Each transfection condition was done in triplicate. Twenty four hours after transfection, one fifth of transfected cells were subjected to transposition assay. The remaining transfected cells (4/5) in triplicate were pooled and grew in a 35-mm plate for another twenty four hours before being subjected to Western blotting. For Western blotting, total proteins were extracted using RIPA buffer (50 mM Tris, pH 8.0, 150 mM NaCl, 1% Nonidet P-40, 0.1% SDS, 0.5% sodium deoxycholate, and 1:100 diluted proteinase inhibitor cocktail) and quantified using the Lowry assay (Biorad). Twenty μg of total proteins were separated by SDS-PAGE on a 8% acrylamide gel. After electrophoresis, the gel were transferred to PVDF membranes (millipore). The membrane was then probed with anti-Myc antibody at 1:1000 (Clontech) and anti-α actin antibody (Calbiochem) at 1:10,000. After three washes, a secondary antibody, peroxidase-conjugated goat anti mouse IgG, was added. After incubation and three washes, the secondary antibodies were subsequently detected by ECL.

### Retrieving chromosomal sequences flanking the transposon targets by plasmid rescue

The same transfection procedure detailed previously was used to transfect the *piggyBac *donor, pXLBacII-cassette, and *Tol2 *donor, Tol2ends-cassette, along with their corresponding helper, pPRIG-*piggyBac *and pPRIG-*Tol2*, respectively, into HEK 293 cells using Fugene HD (Roche). The transposition efficiency for pXLBacII-cassette and Tol2ends-cassette is around 1~2%. To avoid the duplication of the same targeted cell, twenty four hours after the addition of Fugene HD, transfected cells were subjected to a series dilutions and then grown in the hygromycin (100 μg/ml) containing culture medium at a density (about 20 ~ 30 colonies per 100-mm plate as estimated from 1~2% of transposition rate) enabling for isolating individual colonies without cross-contamination. Two weeks after selection, colonies which were at a great distance away from adjacent colonies were individually cloned and expanded until reaching confluence on 100-mm dishes. Genomic DNA of individual clones was isolated and subjected to plasmid rescue. Detailed procedures for plasmid rescue were described previously [32]. Plasmids rescued from the same targeted clone were digested with Hinf II (4-cutter restriction enzyme). For each targeted clone, only plasmids showing different Hinf II digestion patterns were subjected to sequencing. Based on the Hinf II digestion pattern, all of the colonies isolated displayed a distinct repertoire of rescued plasmids indicating that each isolated colony was indeed derived from different targeted cells.

### Q-PCR and Q-RT-PCR

HEK 293 cDNA was obtained using the FastLane Cell cDNA kit (Qiagen). One point three μl of cDNA and 0.125 μg (predetermined by a series dilution of genomic DNA) of HEK 293 genomic DNA were subjected to Q-PCR using primers listed in 2. Q-RT-PCR was performed using SYBR Green PCR Master Mix (Applied Biosystems) in 20 μl of reaction on 7500 Fast Real-Time PCR System (Applied Biosystems). The expression level of individual transcripts was determined by dividing the copy number of each cDNA with the copy number of the corresponding gene using following formula: 2^(Ctgenomic DNA-CtcDNA)^. The relative expression level between each gene and GAPDH was calculated by the ratio of the gene expression level between the two.

### Bioinformatic analyses

Target sites were identified in build hg18 of the human genome using Blat [37], with a sequence identity cutoff of 95%. Human genes were obtained from RefSeq [49], and 2,075 cancer-related genes were taken from the CancerGenes database [50]. Upon counting the number of genes within *n *base intervals, all overlapping genes were first merged to avoid over-counting. CpG islands were taken from the UCSC Genome browser "CpG Island" track, which identifies CpG islands based on the methods of Gardiner-Garden and Frommer [51]. Repeat elements predictions were obtained from RepeatMasker [52]. Only insertions whose first 100 bases are contained within a repeat element were considered to overlap a repeat element. To estimate the significance of the tendency of insertions to be located proximal to CpG islands, we compared the number of insertions located within 2,000 bases of a CpG island to the number expected by chance. The expected number was calculated for each transposon type by picking N random regions in the genome of the same size (in bases) as the given transposon, where N is the total number of insertions for the given transposon. This procedure was repeated 1,000 times, and the mean and standard deviation of the number of random insertions points within 2,000 bases of a CpG island across the 1,000 random trials were used to obtain a Z-score (and associated P-value) for the actual number of insertions located within 2,000 bases of a CpG island.

## Abbreviations

LINE: Long interspersed nuclear element; SINE: Short Interspersed nuclear Element; TRD: Terminal repeat domain; TIR: terminal inverted repeat; PCR: polymerase chain reaction; Q-RT-PCR: Quantitative reverse transcription PCR

## Competing interests

Dr. Yaa-Jyuhn James Meir is partially supported by GenomeFrontier, INC.

## Authors' contributions

YM, and SW participated in experimental design, analysis and drafted the manuscript. MW contributed to the computational analyses and aided in manuscript preparation. PC contributed to the Q-RT-PCR and Q-PCR analyses. HY contributed to processing chromosomal sequence information. RY contributed to the discussion and preparation of the manuscript. All authors read and approved the final manuscript.

## Supplementary Material

Additional file 1 **Table S1**. Clusters of *piggyBac *and *Tol2 *target sites located within a 10 kb interval in HEK 293. A table lists piggyBac and Tol2 targets that are clustered within a 10 Kb interval to the adjacent targets Click here for file

Additional file 2 **Table S2**. A list of primer pairs for Q-RT-PCR analyses. This file contains sequence information for primers used for Quantitative-PCR and Quantitative RT-PC analyses Click here for file
