# Supplementary material for: Genome-wide target profiling of piggyBac and Tol2 in HEK 293: pros and cons for gene discovery and gene therapy
Source: BMC Biotechnol. 2011 Mar 30;11:28. doi: 10.1186/1472-6750-11-28 (PMC3078864; doi:10.1186/1472-6750-11-28)
Supplement: Additional file 1 — Table S1. Clusters of piggyBac and Tol2 target sites located within a 10 kb interval in HEK 293. A table lists piggyBac and Tol2 targets that are clustered within a 10 Kb interval to the adjacent targets [file 1472-6750-11-28-S1.PDF]

Table S1. Clusters of *piggyBac* and *Tol2* target sites located within a 10 Kb interval in HEK 293

Cluster	Target ID	Chromosomal bands	Position	Gene Context	Targeting Gene	Proximal Gene (distance bp)	Distal Gene (distance bp)
A	T22-2	2q21.2	132732800	INTERGENIC		AK0945999 (45962)	BC057764 (96805)
	T145-3	2q21.2	132741835	INTERGENIC		AK094599 (36947)	BC057764 (105840)
	T116-4	2q21.2	132745522	INTERGENIC		AK094599 (33232)	BC057764 (109527)
B	T117-3	6p23	13826113	INTERGENIC		RANBP9 (21006)	BX640678 (68583)
	TB77-2	6p23	13818961	EXONIC	RANBP9		
C	B101-4	10p12.1	25049180	INTRONIC	ARHGAP21		
	TB64-3	10p12.1	25023076	INTRONIC	ARHGAP21		
D	T93-1	18p11.21	12938293	INTRONIC	SEH1L		
	T132-2	18p11.21	12941373	INTRONIC	SEH1L		

Note: The target IDs are *Tol2* in black and *piggyBac* in red.
