# Supplementary material for: Genome-wide target profiling of piggyBac and Tol2 in HEK 293: pros and cons for gene discovery and gene therapy
Source: BMC Biotechnol. 2011 Mar 30;11:28. doi: 10.1186/1472-6750-11-28 (PMC3078864; doi:10.1186/1472-6750-11-28)
Supplement: Additional file 2 — Table S2. A list of primer pairs for Q-RT-PCR analyses. This file contains sequence information for primers used for Quantitative-PCR and Quantitative RT-PC analyses [file 1472-6750-11-28-S2.PDF]

Table S2. A list of primer pairs for Q-RT-PCR analyses

Gene Name	Primer 1	Forward	Primer 2	Reverse
GAPDH	GAPDH F2	CACTGCCACCCAGAAGA	GAPDH R2	GCTTCCCGTTCAGCTCA
MK-1	MK-1 F1	CTGCTCGTTAGCTTTAATCAA	MK-1 R1	CTCTGGGACTCACATT
NRGN	NRGN F1	ACGAGTTCTTTCGTTCTGT	NRGN R1	TCAGAGCAAGGGTCGTC
SYNGR4	SYNGR4 F1	CGGCTACCAGAACAAGATG	SYNGR4 R1	TGTGTGTCCAGGACGAG
FLNB	FLNB F5	CCTCAAGTGCGTGAACAA	FLNB R5	CATTCTCGAGCTGCATCT
MLAS	MLAS F2	CGGAACATCACGCTACTCAA	MLAS R2	GATGGAGAAGACGCCGCA
FLJ32065	FLJ32065 F3	CGGTATTCCGAACAGACACTA	FLJ32065 R3	ATGCAAGGTCCAAAGAGATCA
GPHN	GPHN F4	GTGAGAAAGAATGTGCCAAG	GPHN R4	GGTTCATCATTTTGCGATTGTTTA
SIRPD	SIRPD F3	AACAAACGGAGATGTCACAGA	SIRPD R3	GTAGATTAATTTCCGGTTTGGC
POMZP3	POMZP3 F2	TAATAACACCTCGAAGACGC	POMZP3 R2	TACACACCATCCTGGAGT
GRHL2	GRHL2 F3	GTGAGAAAGAATGTGCCAAG	GRHL2 R3	GGTTCATCATTTTGCGATTGTTTA
ZNF687	ZNF687 F4	CCCAGAGTCCCTCTAGTG	ZNF687 R4	CTGAGTTACAGTCCTTGTGATATT
